# Comparing the Bacterial Diversity of Acute and Chronic Dental Root Canal Infections

**DOI:** 10.1371/journal.pone.0028088

**Published:** 2011-11-21

**Authors:** Adriana L. Santos, José F. Siqueira, Isabela N. Rôças, Ederson C. Jesus, Alexandre S. Rosado, James M. Tiedje

**Affiliations:** 1 Institute of Microbiology Prof. Paulo de Góes, Federal University of Rio de Janeiro, Rio de Janeiro, Brazil; 2 Department of Endodontics and Molecular Microbiology Laboratory, Estácio de Sá University, Rio de Janeiro, Brazil; 3 Center for Microbial Ecology, Michigan State University, East Lansing, Michigan, United States of America; 4 Laboratory of Soil Microbiology, EMBRAPA, Seropédica, Brazil; Argonne National Laboratory, United States of America

## Abstract

This study performed barcoded multiplex pyrosequencing with a 454 FLX instrument to compare the microbiota of dental root canal infections associated with acute (symptomatic) or chronic (asymptomatic) apical periodontitis. Analysis of samples from 9 acute abscesses and 8 chronic infections yielded partial 16S rRNA gene sequences that were taxonomically classified into 916 bacterial species-level operational taxonomic units (OTUs) (at 3% divergence) belonging to 67 genera and 13 phyla. The most abundant phyla in acute infections were *Firmicutes* (52%), *Fusobacteria* (17%) and *Bacteroidetes* (13%), while in chronic infections the dominant were *Firmicutes* (59%), *Bacteroidetes* (14%) and *Actinobacteria* (10%). Members of *Fusobacteria* were much more prevalent in acute (89%) than in chronic cases (50%). The most abundant/prevalent genera in acute infections were *Fusobacterium* and *Parvimonas*. Twenty genera were exclusively detected in acute infections and 18 in chronic infections. Only 18% (n = 165) of the OTUs at 3% divergence were shared by acute and chronic infections. Diversity and richness estimators revealed that acute infections were significantly more diverse than chronic infections. Although a high interindividual variation in bacterial communities was observed, many samples tended to group together according to the type of infection (acute or chronic). This study is one of the most comprehensive in-deep comparisons of the microbiota associated with acute and chronic dental root canal infections and highlights the role of diverse polymicrobial communities as the unit of pathogenicity in acute infections. The overall diversity of endodontic infections as revealed by the pyrosequencing technique was much higher than previously reported for endodontic infections.

## Introduction

Apical periodontitis is a common bacterial biofilm-induced disease that develops around the apex of the dental root and is caused primarily by root canal (endodontic) infection [1]. The disease can manifest itself as different clinical presentations. The asymptomatic (chronic) form is more common and seldom poses a medical problem of significant magnitude, even though evidence is mounting that it contributes to the total oral infectious burden and thus may influence systemic health [2]. Moreover, the typical symptomatic form - the acute apical abscess - can spread from the original site of infection to sinuses and other facial spaces of head and neck and cause serious life-threatening complications [3].

Apical periodontitis has a heterogeneous etiology, where no single species can be considered as the main endodontic pathogen and multiple bacterial combinations play a role in disease causation [4]. Thus far, no strong evidence of the specific involvement of a single species with any particular sign or symptom of apical periodontitis has been found. While some Gram-negative anaerobic bacteria have been suggested to be involved in symptomatic disease [5], [6], [7], the same species are also present in somewhat similar frequencies in asymptomatic cases [8], [9], [10]. Nevertheless, community profiling molecular studies have suggested that the structure of bacterial communities follows specific patterns according to the clinical condition [11], [12]. This suggests that some bacterial community structures may predispose to acute infections instead of the presence of a specific group of species. However, these studies were based on cloning and Sanger sequencing [12], denaturing gradient gel electrophoresis [11] and terminal restriction fragment length polymorphism [12] approaches, all of which are recognized to have the limitation of revealing only the most dominant community members.

Massively parallel DNA pyrosequencing techniques have become widely available over the last years and is now regarded as one of the leading sequencing technologies for 16S rRNA-based bacterial diversity analyses [13]. The technology provides a large number of reads in a single run, resulting in unprecedented greater sampling depth. This allows for detection not only of the dominant community members, but also of the low-abundant microbial populations, the so called “rare biosphere” [13], [14].

Numerous recent studies have used pyrosequencing of 16S rRNA gene to profile the diversity of bacterial communities from diverse environments, including hydrothermal vents of a deep marine biosphere [14], [15] and soil [16], [17]. This technology has also been applied to the analysis of the human microbiota associated with healthy or diseased sites [18], [19], [20], [21], [22], including the oral cavity [23], [24], [25], [26]. These studies disclosed a much larger breadth of bacterial diversity than previously anticipated. So far, only a couple of studies have used this technology to investigate dental root canal infections [27], [28]. However, one was an early study reporting on the ability of the method to unravel the microbiota of 7 infected cases exhibiting different clinical presentations [27], while the other investigated the apical root canal microbiota of teeth with chronic apical periodontitis [28].

Deciphering the composition of the microbiota associated with any infectious disease is of utmost important for a better understanding of the disease pathogenesis and for the establishment of more effective therapeutic protocols. Therefore, the present study was undertaken to evaluate and compare the bacterial diversity of the microbiota associated with acute (abscesses) and chronic dental root canal infections by using a high-throughput multiplexed 16S rRNA gene barcoded pyrosequencing approach.

## Results

Of the pyrosequencing reads that passed the quality control, 13,905 were from acute root canal infections and 13,552 from chronic infections. The average length of the sequences was about 210 bp after trimming the primers.

**Figure 1:**
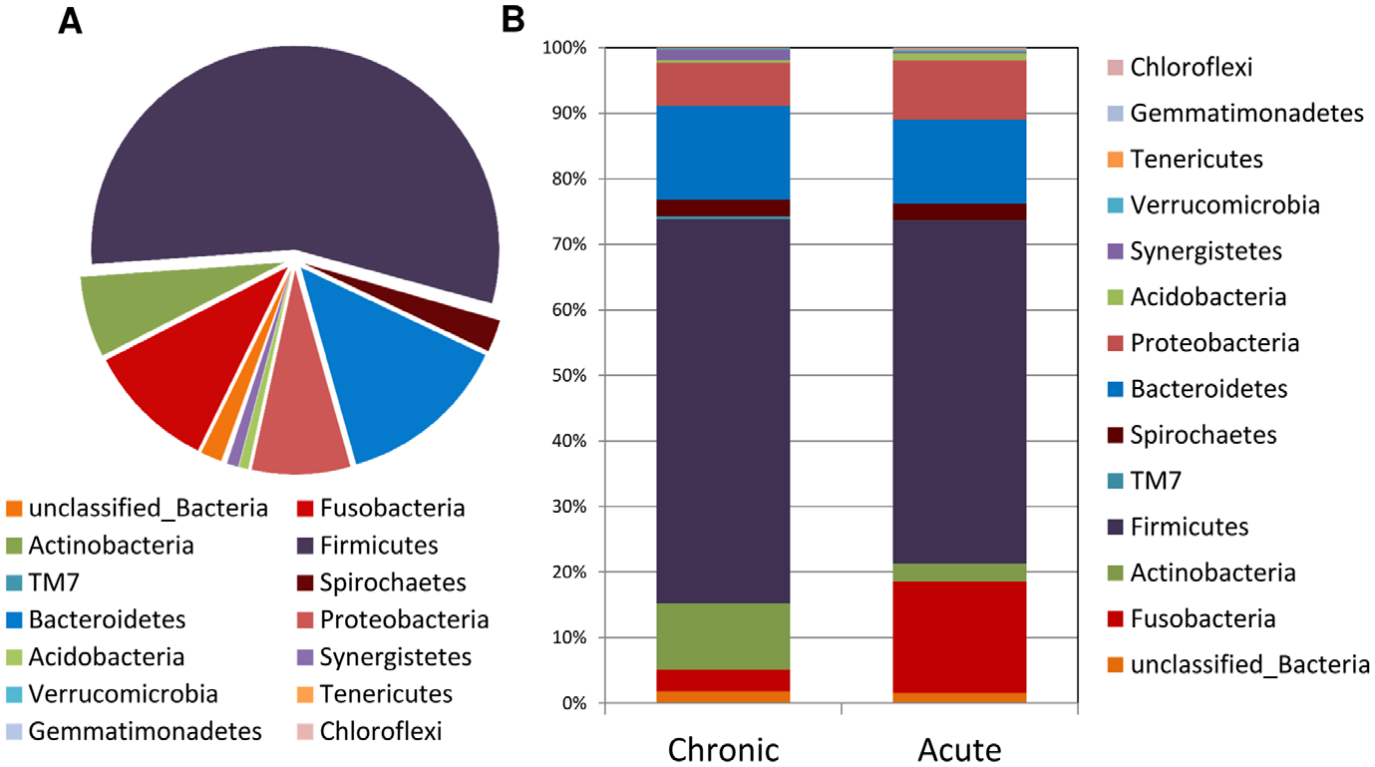
Relative abundance of the different bacterial phyla in acute and chronic dental root canal infections. *A*, overall data. *B,* data according to the clinical condition. Phylogenetic classification was based on Ribosomal Database Project Classifier analyses.

Overall, 13 phyla were represented in endodontic infections (Figure 1). Of the major phyla, *Firmicutes* (52%), *Fusobacteria* (17%) and *Bacteroidetes* (13%) were the most abundant in acute infections, while *Firmicutes* (59%), *Bacteroidetes* (14%) and *Actinobacteria* (10%) were the most abundant in chronic infections (Figure 1). Five of the detected phyla, namely *Firmicutes*, *Bacteroidetes*, *Fusobacteria*, *Actinobacteria* and *Proteobacteria*, collectively constituted more than 90% of the microbiome. Except for *Spirochaetes* (2.6%), each of the other phyla corresponded to less than 1% of the sequences. About 2% of the sequences could not be assigned to any bacterial phylum. In terms of prevalence, members of *Firmicutes* were found in all cases. *Fusobacteria* were much more prevalent in acute (8/9, 89%) than in chronic cases (4/8, 50%). *Bacteroidetes* occurred in 7/9 (78%) acute and 7/8 (87.5%) chronic cases, while representatives of *Actinobacteria* were present in 7/9 (78%) acute and 5/8 (62.5%) chronic cases.

**Figure 2:**
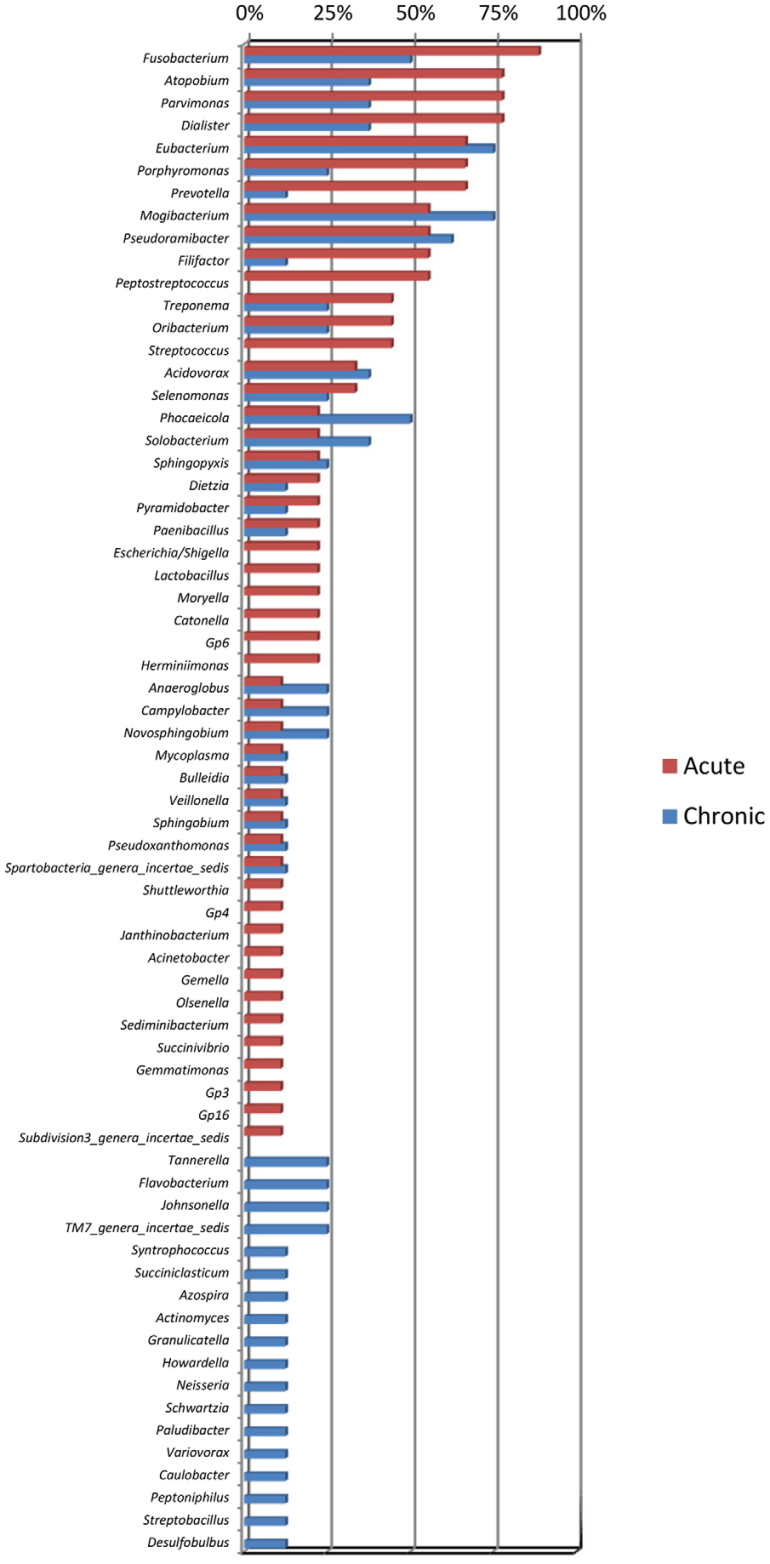
Prevalence of the different genera detected in samples from acute and chronic dental infections.

Overall, sequences were assigned to 67 different genera. Of these, acute and chronic infections were represented by 49 and 47 genera, respectively. The most abundant genera in acute cases were *Fusobacterium* (19%), *Parvimonas* (11%) and *Peptostreptococcus* (10%). *Fusobacterium* was also the most prevalent (8/9, 89%), followed by *Parvimonas, Dialister* and *Atopium* (all detected in 7/9 cases, 78%). Twenty genera were exclusively detected in acute infections, all of them in both low abundance and prevalence. Eleven genera were found in more than 50% of the acute cases (Figure 2).

The most abundant genera in chronic cases were *Phocaeicola* (12.5%), *Eubacterium* (12%) and *Pseudoramibacter* (10%). *Eubacterium* and *Mogibacterium* were the most prevalent (both in 6/8, 75%), followed by *Pseudoramibacter* (5/8, 62.5%) and *Phocaeicola* (4/8, 50%). Eighteen genera were found exclusively in chronic infections, all of them in both low abundance and prevalence. Only 5 genera were found in more than 50% of the chronic cases (Figure 2). About 3% of the sequences could not be classified at the genus level and were placed at the next highest possible resolution level.

**Figure 3:**
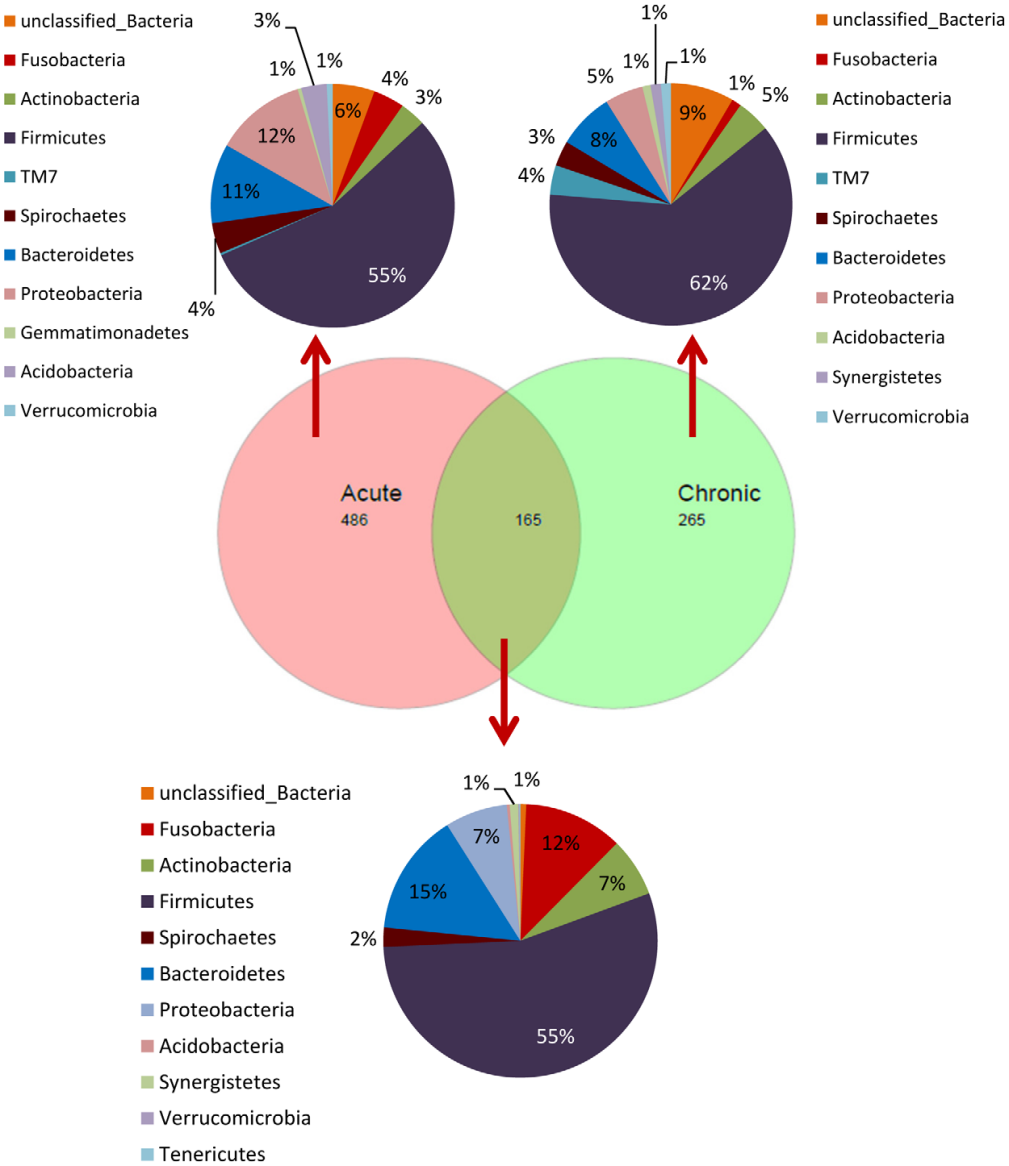
Venn diagram for overlap between observed OTUs at 3% divergence in acute and chronic dental root canal infections. The number of OTUs exclusively found in acute samples was 486 and in chronic samples was 265. The number of OTUs shared between acute and chronic infections was 165. Percentage of shared OTUs was 18%. Data are also represented by the phyla which the detected OTUs belong to. Data regarding genera are shown as supplementary material.

**Figure 4:**
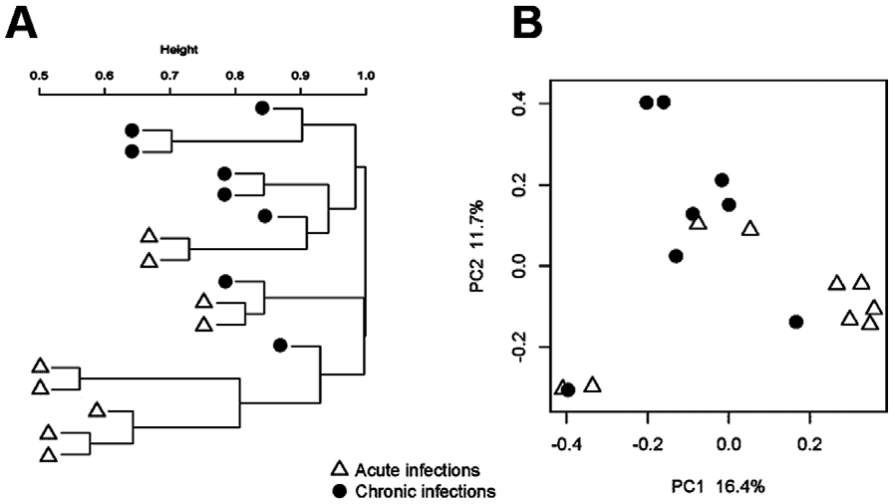
Cluster (A) and PCoA (B) analyses of acute (symptomatic) and chronic (asymptomatic) dental root canal infections. Although a high interindividual variability can be observed, some samples tended to group together according to the type of infection.

For OTUs at 3% distance (species level), 916 different phylotypes were detected. Of these, 651 phylotypes were found in acute cases and 430 in chronic cases. The percentage of species-level taxa shared in acute and chronic groups was 18% (165 species) (Figure 3). The mean number of OTUs at the 3% dissimilarity level present per acute case was 114 (range, 56 to 225) compared to 71 OTUs (range, 42 to 104) in chronic cases. Data regarding shared genera are available as supporting information (Figure S1). Table 1 depicts data from diversity and richness estimate calculations. Calculation of Shannon estimator of diversity at 3% difference revealed that acute infections were significantly more diverse than chronic infections, with no overlap of the 95% CIs. Using the ACE nonparametric estimator of richness, it was possible to observe that there are a predicted 1,466 species-level OTUs in the acute cases and 1,031 in the chronic cases. Based upon Chao1, there is an average of 1,090 and 857 species-level OTUs in acute and chronic infections, respectively. The shapes of the rarefaction curves confirmed that acute infections are more diverse than chronic infections (Figure S2). Rarefaction curves also indicated that bacterial richness in acute and chronic infections is not yet completely revealed by the number of sequences analyzed. Although a high interindividual variation in bacterial communities was revealed by PcoA and cluster analyses, there was a trend for many samples to group together according to the type of infection (acute or chronic) (Figure 4).

**Table 1:** Sequencing data and diversity estimate calculations for bacterial taxa in acute and chronic dental root canal infections.

	Acute	Chronic
Total number of sequences	13,905	13,552
Total OTUs at 3% difference (phylotypes)	676	443
Total OTUs at 5% difference (phylotypes)	555	352
Shannon estimator at 3% difference (95% CI)	4.12 (4.09; 4.15)	3.95 (3.92; 3.97)
Shannon estimator at 5% difference (95% CI)	3.86 (3.83; 3.89)	3.85 (3.83; 3.88)
Chao1 estimator of richness at 3% (95% CI)	1,090 (972; 1,250)	857 (712; 1,072)
Chao1 estimator of richness at 5% (95% CI)	922 (819; 1,065)	668 (554; 844)
ACE estimator of richness at 3% (95% CI)	1,466 (1,342; 1,612)	1,031 (921; 1,165)
ACE estimator of richness at 5% (95% CI)	1,184 (1,081; 1,308)	800 (710; 913)
ESC [Cx = 1 - (Nx/n); Nx = unique sequences/n = total sequences]	0,62	0,65

## Discussion

The present findings indicate that there is a significantly higher diversity of bacteria in acute dental infections (abscesses) when compared to asymptomatic chronic infections. This significantly increased diversity may be an important aspect of acute infections and the possibility exists that the microbiota present in these cases may contain harmful bacterial species contributing to the severity of symptoms. Also, as a highly diverse polymicrobial infection, incalculable synergistic interactions between multiple bacterial species are expected and can result in increased pathogenicity. Therefore, the ability of the community to cause disease is very likely to be related to collective pathogenicity and is coherent with the current trend to categorize the bacterial community as the unit of pathogenicity for many endogenous diseases [29], [30], [31].

A recurrent theme in endodontic microbiology research is the desire to find the major pathogen responsible for acute disease. This study failed to disclose a single specific taxon associated with acute infections. Actually, many bacterial taxa were either exclusive or much more prevalent/abundant in acute infections than in chronic cases. Most of these taxa were rather in low abundance and may have passed unnoticed in previous culture and molecular studies. The present results suggest that the composition of the bacterial community can be much more important to the development of acute symptoms than the mere presence of a potentially pathogenic species. It is also possible to speculate that those species found in higher prevalences or exclusively in acute cases play a decisive ecological role in determining the virulence of the consortium. Because none of these taxa were found in all cases, the possibility of functional redundancy in the pathogenic community is suspected.

Previous studies comparing acute and chronic infections have suggested that some species are more related to acute symptoms [5], [6], [7], [32], more species are found per individual acute case than per chronic case [11], [12], and some community profiles are more related to disease severity [11], [12]. Most of these findings were also evident and expanded in the present study using deep-coverage pyrosequencing. Taken together, these data reveal that acute infections have a more complex microbiota and interactions between the numerous community members may be critical for the development of symptoms.

A marked interindividual variability in the composition of the bacterial communities was observed. Each individual harbored a unique endodontic microbiota in terms of species richness and abundance. This is in agreement with previous molecular studies using community profiling techniques [11], [33], [34]. The fact that the composition of the microbiota differs consistently between individuals suffering from the same disease denotes a heterogeneous etiology for apical periodontitis, where multiple communities can lead to similar disease outcomes. Despite this interindividual variability, most samples showed a tendency to group together according to the presence of symptoms. This suggests that there may exist some patterns of community structures related to distinct clinical conditions.

Our overall findings revealed 67 genera belonging to 13 phyla in primary endodontic infections. A recent comprehensive compilation of findings from previous culture and molecular studies demonstrated that more than 460 bacterial species/phylotypes belonging to 100 genera and 9 phyla have been detected in the different types of endodontic infections [35]. The present findings strongly indicate that these numbers may have been grossly underestimated. Application of barcoded parallel pyrosequencing to the study of endodontic infections provided a view of the bacterial diversity associated with apical periodontitis at a much deeper level. This is in consonance with an early study of root canal infections using pyrosequencing [27]. Nonetheless, irrespective of the depth of analysis, true diversity was still greater than that identified in this study, as revealed by diversity and richness estimators and rarefaction curves.

Of the 13 phyla represented in this study, *Verrucomicrobia* and *Gemmatimonadetes* had not been previously reported in endodontic infections. Of the major phyla, *Firmicutes* and *Bacteroidetes* were the most abundant and prevalent, which is in agreement with previous studies using culture methods or cloning and Sanger sequencing [12], [36], [37]. Noteworthy was the fact that members of the *Fusobacteria* phylum were much more adundant and prevalent in acute than in chronic infections. A species of this phylum – *Fusobacterium nucleatum* – has been frequently identified in acute endodontic infections [7], [38], [39], [40].

The vast majority of the species-level phylotypes occurred at very low levels. This confirms the great potential of pyrosequencing analysis to reveal the rare biosphere. At this stage, it is not possible to infer a role for these bacteria in the community. However, it is widely recognized in microbial ecology that even low-abundant members might serve as keystone species within complex communities [41], [42]. Low-abundant members may hold the potential to become dominant in response to shifts in environmental conditions [41]. Finally, a consistent understanding of the ecology and pathogenicity of a microbial community requires the thorough knowledge of every component involved, including identification of species present at low levels in the environment [29].

It has been shown that factors such as the number of sequences analyzed and the sample size can influence the species richness and the overall diversity [43], [44]. Therefore, one must assume that microbial community analyses based on the traditional cloning and Sanger sequencing are limited to identification of the most abundant taxa in a sample. The greatest advantage of the pyrosequencing approach over the traditional cloning and sequencing method is that a much larger number of 16S rRNA sequence reads can be obtained in a single run, providing a huge coverage depth. Moreover, the pyrosequencing approach also avoids the biases inherent to the cloning procedure. Nevertheless, the short length of reads generated by this high-throughput technology may represent a limitation in terms of bacterial taxonomy characterization. Even so, it has been shown that reads spanning particular variable regions of the 16S rRNA gene can still be highly informative and that despite the shorter read lengths, the pyrosequencing approach provides a description of the microbiome that is in good agreement with that provided by the cloning and Sanger sequencing approach [25], [45]. In order to avoid overestimates when analyzing short sequence reads, we abided by the recommendations of Kunin et al. [13], who recommended a stringent quality-based trimming of the reads and a cut-off value for identification no greater than 97%. However, about 2% of bacterial taxa were unclassified, which can be considered as high at the phylum level. The possibility exists that this may be probably not due to unknown bacterial phyla, but to sequencing errors, short sequencing reads or PCR artifacts.

In conclusion, our findings using the massive parallel pyrosequencing analysis of dental root canal samples revealed that the bacterial diversity associated with acute infections is higher than chronic infections. It is also reasonable to conclude that the severity of disease (intensity of signs and symptoms) may be related to the bacterial community composition. This means that the disease outcome is a result of a summation of attributes of a pathogenic community. Further studies evaluating the activity and pathogenic potential of the endodontic bacterial communities should be encouraged using methods such as proteomics, transcriptomics and metabolomics. The overall diversity of endodontic infections as revealed by the pyrosequencing technique was much higher than previously anticipated.

## Materials and Methods

### Case description, sample taking and DNA extraction

The study protocol was approved by the Ethics Committee of the Estácio de Sá University, Rio de Janeiro, and written informed consent was obtained from the patients. Samples were taken from 17 patients who had been referred for root canal treatment or emergency treatment to the Department of Endodontics, Estácio de Sá University. Only single-rooted teeth from adult patients (ages ranging from 18 to 62 years), all of them having carious lesions, necrotic pulps and radiographic evidence of apical periodontitis (bone destruction around the dental root apex), were included in the study. Selected teeth showed an absence of periodontal pockets deeper than 4 mm. Eight asymptomatic cases were diagnosed as chronic apical periodontitis and nine cases were diagnosed as acute apical abscesses. Diagnosis of acute apical abscess was based on the presence of spontaneous pain, exacerbated by mastication, and localized or diffuse swelling, along with fever, lymphadenopathy, or malaise.

In cases of chronic apical periodontitis, samples were obtained from the root canals under strict aseptic conditions, which included rubber dam isolation and a two-step disinfection protocol of the operative field with 2.5% NaOCl as previously described [11]. Endodontic files with the handle cut off and paper points used for sampling the root canals were transferred to cryotubes containing TE buffer (10 mM Tris-HCl, 1 mM EDTA, pH 7.6) and immediately frozen at –20°C. Abscesses were sampled by aspiration of the purulent exudate from the swollen mucosa over each abscess. The overlying mucosa was disinfected with 2% chlorhexidine solution, and a sterile disposable syringe was used to aspirate pus, which was immediately injected into cryotubes containing TE buffer and frozen at −20°C. DNA was extracted from clinical samples using the QIAamp DNA Mini Kit (Qiagen, Valencia, CA) according to the manufacturer's instructions. To maximize DNA extraction from Gram-positive bacteria, a step of pre-incubation with lysozyme for 30 min was added.

### Pyrosequencing

Partial 16S rRNA gene sequences were amplified from clinical samples using the barcoded-primer approach to multiplex pyrosequencing. Polymerase chain reaction (PCR) amplification of the V4 region of the 16S rRNA gene was performed using 8-bp barcoded degenerate eubacterial primers 563F and 802R (http://pyro.cme.msu.edu/pyro/help.jsp). PCR mixtures were as described elsewhere [17]. Equimolar amplicon suspensions were combined and subjected to pyrosequencing using a Genome Sequencer FLX system (454 Life Sciences, Branford, CT) at the Michigan State University Genomics Technology Support Facility.

### Sequence processing and statistical analysis

Raw sequences were processed through the Ribosomal Database Project (RDP) pyrosequencing pipeline (http://wildpigeon.cme.msu.edu/pyro/index.jsp). Sequences were excluded from the analysis if the read length was less than 150 bp, if the minimum average exponential quality score was lower than 20 (Average Qscore of 20 for 16S sequences) or if the primer sequences contained errors (about 13%). Qualified sequences were clustered into operational taxonomic units (OTUs) defined by a 3% distance level using complete-linkage clustering and these were assigned to phyla using the RDP-II classifier at a 50% confidence threshold [46]. The sequences obtained in this study were uploaded and are availableat the Sequence Read Archive (SRA) at URLhttp://www.ebi.ac.uk/ena/data/view/ERP000669. Sequences that could not be classified into a phylum at this level of confidence were excluded from subsequent phylum composition analyses.

A total of 27,457 partial 16S rRNA sequences were obtained from the clinical samples. Phylum composition was determined by taxonomic assignment performed by Classifier [46] with default parameters via the RDP II web site. Multiple sequence alignments for each sample were performed with Infernal Aligner (with the default parameters) via RDP II web site [47]. Based on the alignment, a distance matrix was constructed by the Mothur v 1.17.3 package [48] with the default parameters using the Jukes-Cantor model option [49]. These pairwise distances served as inputs for clustering the sequences into OTUs. The clusters were made at a 3% dissimilarity cut off and served as OTUs for generating predictive rarefaction models and for making calculations of the richness indices Ace and Chao1 [50] and Shannon's diversity index [51]. These analyses were made using Mothur v 1.17.3 package [48]. A 3% distance-level OTU matrix was used to calculate a distance matrix with the Bray-Curtis distance. This matrix was submitted to clustering and principal coordinates analysis (PCoA) according to Kindt and Coe [52]. The algorithm used for clustering was the complete linkage algorithm. These analyses were performed with the package vegan [53] for program R (http://www.R-project.org/).

## Supporting Information

Figure S1 Venn diagram for overlap between observed OTUs at the genus level in acute and chronic dental root canal infections.
(TIF)

Figure S2 Rarefaction curves used to estimate richness of acute and chronic dental root canal infections. The vertical axis shows the number of OTUs at 3% and 5% divergence expected to be disclosed after sampling the number of sequences shown on the horizontal axis.
(TIF)
