# Supplementary figures and images for: Comparing the Bacterial Diversity of Acute and Chronic Dental Root Canal Infections
Source: PLoS One. 2011 Nov 21;6(11):e28088. doi: 10.1371/journal.pone.0028088 (PMC3221700; doi:10.1371/journal.pone.0028088)

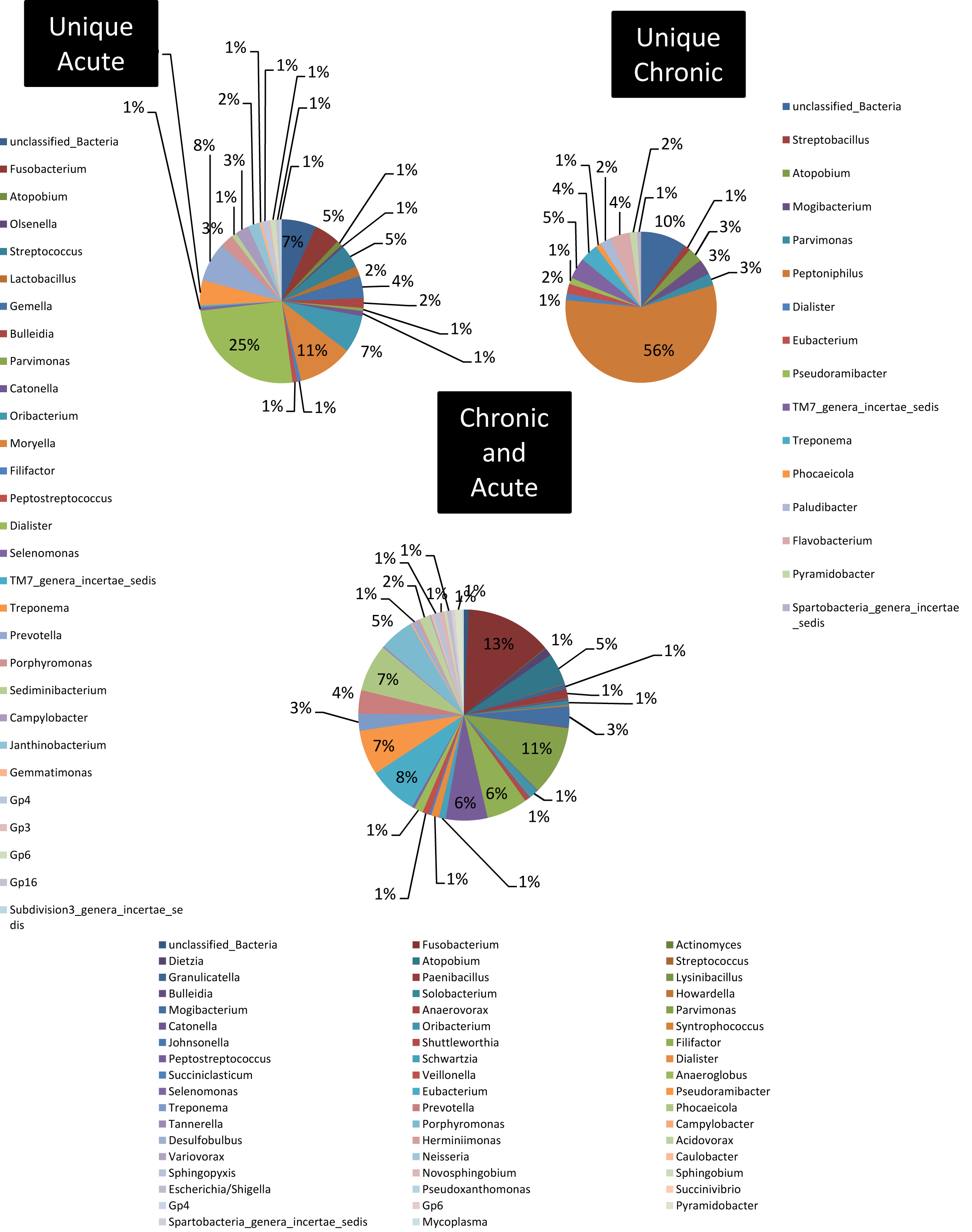

Supplement: Figure S1 — Venn diagram for overlap between observed OTUs at the genus level in acute and chronic dental root canal infections. (TIF) [file pone.0028088.s001.tif]

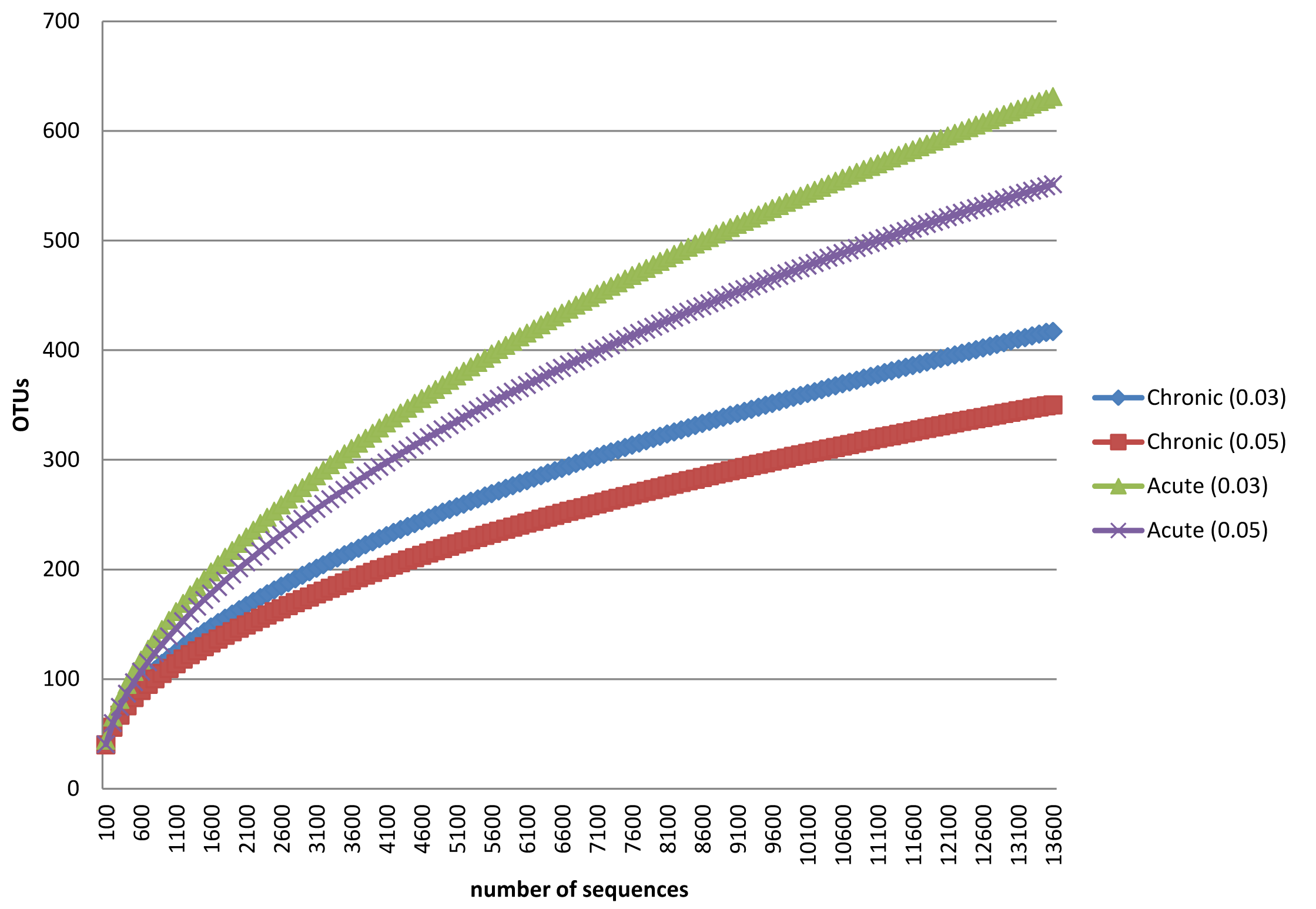

Supplement: Figure S2 — Rarefaction curves used to estimate richness of acute and chronic dental root canal infections. The vertical axis shows the number of OTUs at 3% and 5% divergence expected to be disclosed after sampling the number of sequences shown on the horizontal axis. (TIF) [file pone.0028088.s002.tif]
